# Supplementary material for: Physiological Analysis and Transcriptome Profiling of Inverted Cuttings of Populus yunnanensis Reveal That Cell Wall Metabolism Plays a Crucial Role in Responding to Inversion
Source: Genes (Basel). 2018 Nov 23;9(12):572. doi: 10.3390/genes9120572 (PMC6316517; doi:10.3390/genes9120572)
Supplement: Supplementary file 1 [file genes-09-00572-s001.pdf]

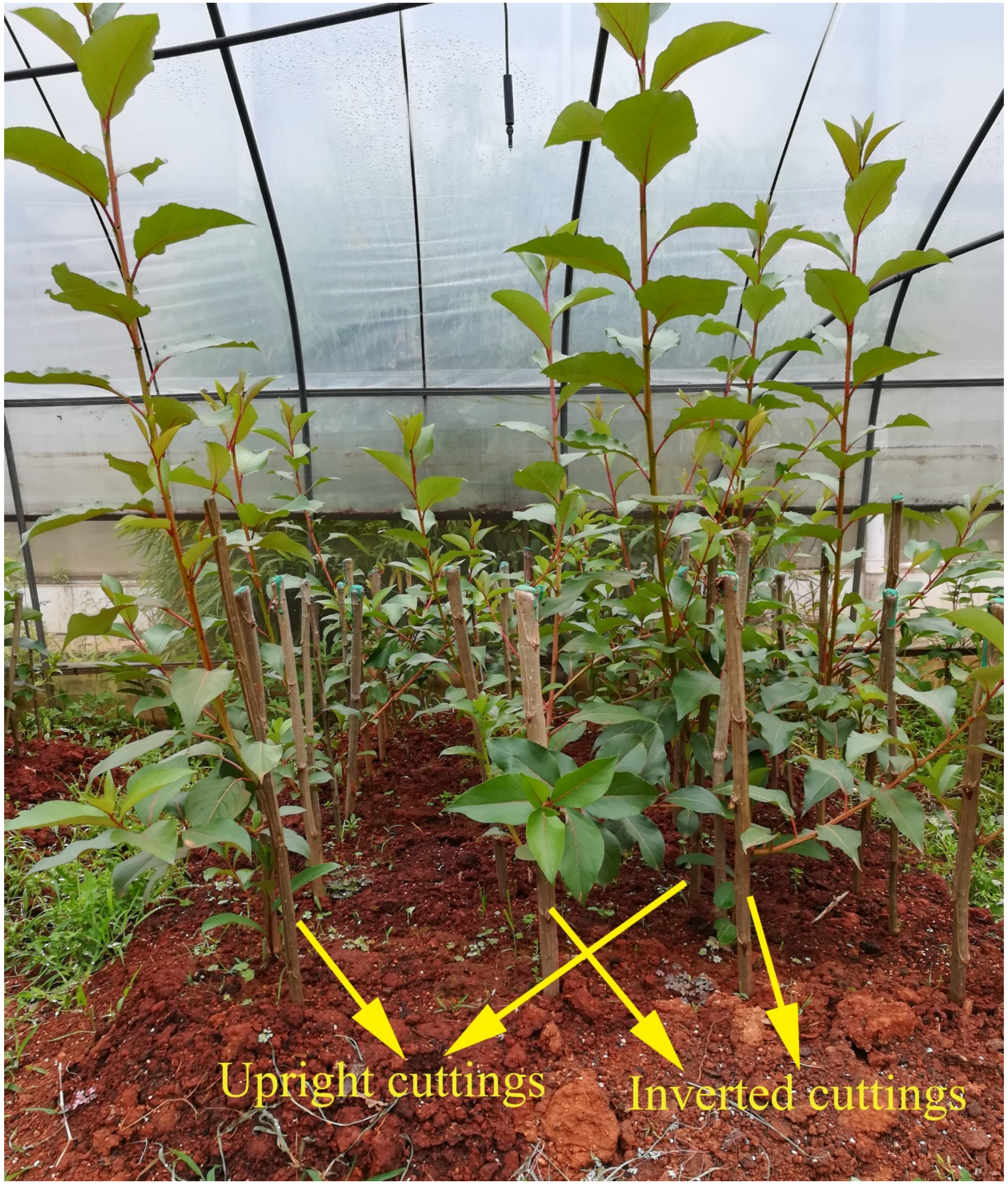
Upright cuttings
Inverted cuttings

Figure S1. Vegetative form of upright and inverted cuttings of *P. yunnanensis*.

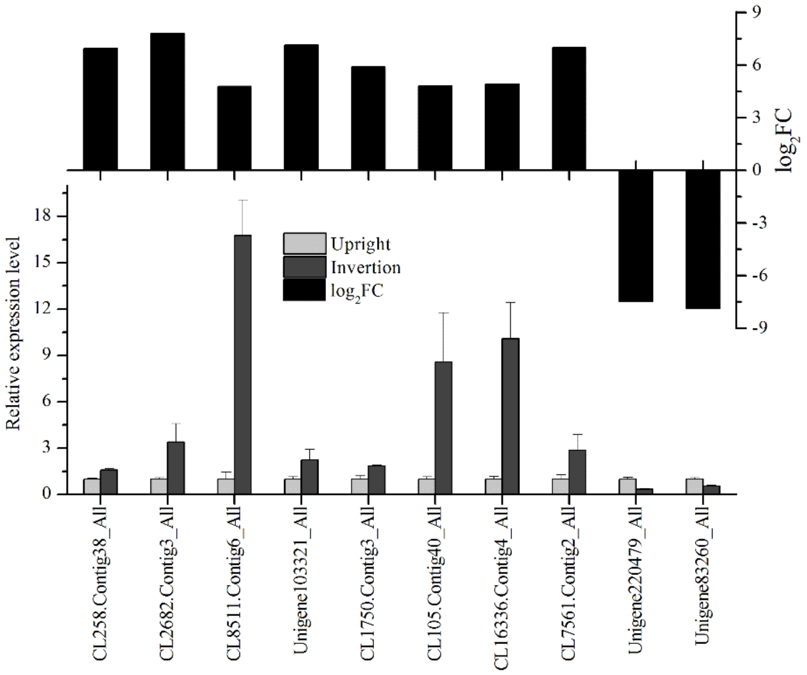

Figure S2. Confirmation of RNA-Seq results using RT-qPCR.

Table S1. Information for ten selected DEGs.

Unigene	Length	KO ID	KEGG pathway map	KEGG annotation
CL258.Contig38_All	1853	K00963	map 00052; map 00500; map 00040; map 00520	UTP--glucose-1-phosphate uridylyltransferase
CL2682.Contig3_All	1504	K01092	map 00562	myo-inositol-1(or 4)-monophosphatase

CL8511.Contig6_All	2510	K09840	map 00906	9-cis-epoxycarotenoid dioxygenase
Unigene103321_All	510	K01051	map 00040	pectinesterase
CL1750.Contig3_All	3567	K06617	map 00052	raffinose synthase
CL105.Contig40_All	1020	K18819	map 00052	inositol 3-alpha-galactosyltransferase
CL16336.Contig4_All	3385	K06611	map 00052	stachyose synthetase
CL7561.Contig2_All	463	K01836	map 00052	phosphoacetylglucosamine mutase
Unigene220479_All	246	K12447	map 00040; map 00052; map 00053; map 00520	UDP-sugar pyrophosphorylase
Unigene83260_All	231	K12373	map 00511; map 00513; map 00520; map 00531; map 00603; map 00604; map 04142	hexosaminidase
